# Investigation of the reasons and frequency of oncology patients over the age of 65 who apply to the emergency department

**DOI:** 10.1007/s00520-025-09462-1

**Published:** 2025-04-25

**Authors:** Nedime Hazal Döner, Öznur Usta Yeşilbalkan, Uğur Taktuk

**Affiliations:** 1 Izmir University of Economics, Vocational School of Health Services, Balçova, , İzmir, 35330 Turkey; 2 Faculty of Nursing, Department of Internal Medicine Nursing, Ege University, Izmir, Turkey; 3 Department of Emergency Medicine, Ege University Hospital, Ege University, Izmir, Turkey

**Keywords:** Emergency service, Oncology, Older people, Cancer care

## Abstract

**Purpose:**

This study aims to investigate frequency and causes of emergency department visits among oncology patients aged 65 and older.

**Method:**

In this cross-sectional, descriptive and comparative study, data were collected a questionnaire form from a single emergency deparment of a university hospital in İzmir, West Turkey between January 2022 and July 2022. Data were analyzed by using IBM SPSS Statistics 25 software.

**Results:**

A total 125 patients were included in the study. The most common causes of patients to the emergency derparment were infection, pain, nausea-vomiting, and dyspnea. A significant difference was found between the reason for the patient’s emergency department visit and their knowledge of when to seek medical help (*p* < 0.05).

**Conclusion:**

The recurrent visits of cancer patients to emergency departments suggest an inadequacy of comprehensive information available to both patients and their caregivers concerning the pursuit of emergency medical intervention.

## Introduction

Aging is considered a physiological process that leads to various changes in organs and systems, causing physical, cognitive, and emotional transformations in individuals [1, 2]. In recent years, advancements in technology and healthcare have resulted in an increased lifespan [3]. These positive developments have contributed to a higher proportion of the elderly within the overall demographic [4].

World Health Organization (WHO) considers individuals aged 65 and above as elderly [5, 6]. The number of this age group worldwide reached 727 million in the year 2020 [7]. Based on this information, it is projected that by the year 2050, this group will constitute approximately 16.7% of the world’s population [8]. The number of individuals aged 65 and over in Turkey exceeded 5.7 million in 2012. It is estimated that this figure will reach 19.5 million in 2050 in Turkey as life expectancy increases [9, 10].

Despite all advancements in the diagnosis and treatment of cancer, this disease still leads to chronic pain and premature death. Cancer is a major cause of death in the world; in Turkey, it ranks second after cardiovascular diseases [11]. With the increasing elderly population, there has been an increase in the number of new cancer cases and the associated side effects of treatment [12].

Due to the increase in the number of chronic diseases and the decrease in physical capacity with age, there are more elderly patients applying to emergency services than younger adults. The literature shows that the emergency department (ED) admission rates of elderly patients were between 12 and 24% [13]. An examination of studies on ED applications for all reasons in Turkey reveals the following rates for this group: in the study by Bilgili and Öncü, the ED application rate of elderly patients is 10.1%; In the study by Unsal et al. in Eskişehir, the application rate was 13%; in the study by Kekeç et al., 14.3%; and in the study by Çığsar et al., 19.6% [5, 14–16].

The widespread adoption of early diagnosis and treatment systems and the advancement of technology has led to new treatment methods. The increasing number of cancer cases and novel treatment approaches result in a longer monitoring period, leading to more frequent ED visits [5]. The extension of follow-up periods due to increased life expectancies results in a higher incidence of local and systemic complications caused by cancer, as well as complications related to treatment. Therefore, cancer patients constitute a patient group that significantly increases Eds’ workload and presents unique psychosocial challenges [17].

Although the reasons for elderly patients’ admission to the ED vary, according to Hu et al., the main reasons were reported to be cerebrovascular events, oncological diseases, and cardiovascular diseases [18]. Among studies in Turkey the study by Bedel and Tomruk reveals that leading reasons are cardiovascular diseases and oncological diseases [19]. Studies show that 81% of ED visits of elderly patients are related to oncology [20, 21]. However, no study in Turkey was found that investigated the reasons and frequency of admission to the ED of oncology patients aged 65 and over.

It is crucial to determine the health status of elderly patients with advanced-stage cancer because treatment benefits are often limited, and treatment toxicity is high [22]. Consequently, elderly patients present to EDs have more complex issues, require more intensive services, and undergo a greater number of diagnostic procedures. Additionally, their hospitalization and admission rates to intensive care are higher compared to other age groups [23]. The increasing prevalence of geriatric oncology patients in EDs necessitates facilities with the appropriate physical conditions and higher levels of qualified care.

In this study, It was aimed to investigate the reasons and frequency of admission of oncology patients over the age of 65 who applied to the ED. The sub-goals of the study are as follows: to identify from these applicants the geriatric patients whose applications were recorded as malignant neoplastic diseases, to determine the clinical evaluation and reasons of oncology-related complaints, to investigate whether they received information about the number of applications, the length of stay in the ED, and the clinical complaints for which they would apply to the emergency room.

## Material-method

### Study design

This study used a cross-sectional, a descriptive, and a comparative study design.

### Sample, setting, and ethic considerations

This cross-sectional, descriptive, and comparative study was carried out from January 2022 and July 2022 in the emergency deparment of a large university hospital in Izmir, West Turkey. A total of 125 patients participated in the study. The study was approved by the ethics committees of the Ethics Committee (Approval Date: 28.01.2022, Approval No: 22–1.1 T/16). Institutional permission for data collection was obtained from the Emergency Department of University Hospital (Approval Date: 20.12.2021, Reference No: E- 27344949–100–465304).

### Procedure

The purpose of the study was explained to the participants, and then all participants signed a written consent form prior to participation. A convenience sample of patients was obtained from among all patients who were visiting the emergency department. Patients were included if they met the following criteria: (a) 65 years of age or older, (b) have a history of cancer, (c) at least once received cancer treatment, (d) ability to speak, read, and write in Turkish, (e) no auditory or visual impairment, and (f) willingness to participate in the study. The data was collected face to face by researchers.

### Instruments

The data were collected by using a questonnaire form. This questionnaire form consisted of 19 questions and included socio-demographics (age, gender, marital status, educational status, economic status, and whom they live with—***six question***) and disease-related characterictics (chronic illness status, type of cancer, cancer treatment administered, number of treatments received, most recent treatment time, and information received regarding when to seek emergency department care after treatment—***seven question***), and reason for emergency department visit, actions taken to relieve symptoms before arrival, previous reasons for emergency visits, number of emergency visits in the past year, duration of observation in the emergency department, and decision made after arrival (e.g., admission, discharge) (***six question***). This form was also developed by the researcher according to the literature and consists of 19 questions. This form was pretested on ten patients in order to check the clarity of the items, and no changes were recommended. The researcher read the questionnaire items to patients and recorded their responses.

### Data analysis

The researcher provides the descriptive statistics of the data (number, percentage, mean, standard deviation, minimum, and maximum). The first step in data analysis was to check the assumption of normality using the Shapiro–Wilk test. In cases where this assumption was not met, the Mann–Whitney *U* test was conducted to examine the difference in means between independent groups. When the assumption of sample size was not met for examining relationships between categorical variables (expected value < 5 for each group), Fisher’s exact test was used. The Chi-square test was employed for investigating the relationship between multiple-choice questions. The analyses were conducted using IBM SPSS Statistics 25 software.

## Results

### Socio-demographic characteristics of participants

Examination of the demographic characteristics of the participants shows that the average age was 70.92 ± 3.47 years. Of the participants, 52% were male; 92.8% were married; 55.2% had primary/middle school education; 51.2% had an income less than their expenses; and 8.8% lived alone (Table 1).

**Table 1:** Distribution of participants according to demographic characteristics

	*n*	Min		Max	Mean		S.D
**Age**	125	64		96	70.92		6.47
						*n*	%
**Gender**			Female			60	48.0
			Male			65	52.0
**Marital status**			Single			9	7.2
			Maried			116	92.8
**Educational status**			Primary/secondary school			69	55.2
			High school			23	18.4
			University			28	22.4
			Degree			5	4.0
**Income status**			Income less than expenses			64	51.2
			Income equals expenses			47	37.6
			Income exceeds expenses			14	11.2
**Person living with**			Alone			14	11.2
			Spouse/family			111	88.8

### Disease-related findings

The three most common hematologic/oncologic diagnoses of the participants presenting to the ED were lung cancer (14.4%), breast cancer (13.6%), and stomach cancer (10.4%); 82.4% of the participants (103 individuals) reported receiving chemotherapy before, and 24.8% of the participants who had received chemotherapy had undergone six to ten sessions. When questioned about the conditions under which they should visit the ED before treatment, it was found that 60.8% of participants (76 individuals) had no information (Table 2).

**Table 2:** Distribution of participants according to disease-related charecteristics

		*n*	%
**Chronic disease**	Yes	75	60
	No	50	40
**Type of cancer**	Lung cancer	18	14.4
	Prostate cancer	10	8.0
	Pancreatic cancer	10	8.0
	Lymphoma/leukemia	13	10.4
	Breast cancer	17	13.6
	Thyroid cancer	3	2.4
	Colorectal cancer	12	9.6
	Gastric cancer	13	10.4
	Ovarian cancer	4	3.2
	Skin cancer	2	1.6
	Sarcoma	3	2.4
	Glioma	6	4.8
	Larynx/nasopharynx cancer	3	2.4
	Endometrial cancer	2	1.6
	Cervical cancer	1	0.8
	Multiple myeloma	3	2.4
	Bladder cancer	3	2.4
	Hepatocellular cancer	2	1.6
**Cancer treatment administered**	Chemotherapy	103	82.4
	Radiotherapy	52	41.6
	Surgical	74	59.2
	Oral chemotherapy/immunotherapy	5	4.0
	Patients for whom no intervention is planned	3	2.4
**Number of treatments received**	1 time	6	4.8
	2–5 times	28	22.4
	6–10 times	31	24.8
	11–20 times	22	17.6
	21 times or more	15	12.0
**Information received regarding when to seek emergency department care after treatment**	Yes	49	39.2
	No	76	60.8

## Findings regarding emergency department admissions

The four most common reasons for applying to the ED are infection, pain, nausea-vomiting, and dyspnea (Table 3). It was determined that most participants presenting with infection were in the grade 2 category, those with pain complaints, in the grade 1 category, those with dyspnea complaints, in the grade 1 category, and those with nausea and vomiting, in the grade 2 category.

**Table 3:** Distribution of participants according to the reasons for ED visits

		*n*	%
**Reason for emergency department visit**	Infection	57	45.6
	Pain	46	36.8
	Nausea-vomiting	35	28.0
	Dyspnea	34	27.2
	Tiredness	26	20.8
	Electrolyte imbalance	16	9.7
	General condition disorder/oral intake disorder	15	12.0
	Constipation	13	10.4
	Neutropenic fever	9	7.2
	Diarrhea	8	6.4
	Anemia	8	6.4
	Neutropenia	6	4.8
	Dysuria	5	4.0
	Thrombocytopenia	5	4.0
	Hematuria	3	2.4
	Mucositis	2	1.6
	Other	2	1.6

When questioned about any interventions before arriving at the ED, 72.8% (91 individuals) reported making no interventions to relieve their symptoms. Of the others, participants reported using antiemetic drugs (29.4%) to alleviate nausea and vomiting, analgesic drugs (44.1%) to alleviate pain complaints, and oxygen (14.7%) to alleviate dyspnea symptoms.

The findings indicate that 89.6% of the participants (112 individuals) sought ED care between 1 and 3 times in the last year; 56% (70 individuals) were monitored for 1–3 days. There was a statistically significant relationship between participants’ reasons for ED visits and their level of knowledge about when to seek assistance (*p* < 0.05).

It was found that information was not usually received for people with nausea-vomiting, electrolyte imbalance, general condition disorder, oral intake disorder, fatigue, pain, anemia, and diarrhea, but most people with neutropenia and neutropenic fever receive information. Multiple Chi Square test was performed to examine the relationships between hematological and oncological diagnoses and the reasons for applying to the ED, revealing a statistically significant relationship between the diagnoses received and the reasons for applying (*p* < 0.05).

### Comparison of the relationships between participants' demographic characteristics and the number of ED visits

A statistically significant relationship was found between the number of ED visits and gender (*p* < 0.05). It was determined that men had a greater frequency of ED visits than women. A statistically significant relationship was found between the participants’ household and the number of visits to the emergency room (*p* < 0.05). It was determined that most of those living alone or with their spouse/family applied to the ED 1–3 times, and most of those living with others (caregiver, in a nursing home, etc.) applied 4–9 times.

There was no statistically significant relationship between the number of ED visits and marital status, educational level, income status, and chronic disease status (*p* > 0.05). The analysis result showed no statistically significant difference in age averages based on the number of ED visits (*p* > 0.05) (Table 4).

**Table 4:** Comparison of the relationships between participants’ demographic characteristics and the number of ED visits

			Number of applications				
			1–3 times	4–9 times	10 times or more	Test statistics	*p*
**Gender**	Female	*n*	58	2	0	6.201	0.02*
		%	96.7	3.3	0.0		
	Male	*n*	54	10	1		
		%	83.1	15.4	1.5		
**Marital status**	Single	*n*	6	3	0	6.099	0.11
		%	66.7	33.3	0.0		
	Married	*n*	106	9	1		
		%	91.4	7.8	0.9		
**Educational status**	Primary/secondary school	*n*	62	6	1	4.584	0.77
		%	89.9	8.7	1.4		
	High school	*n*	20	3	0		
		%	87.0	13.0	0.0		
	University	*n*	26	2	0		
		%	92.9	7.1	0.0		
	Degree	*n*	4	1	0		
		%	80.0	20.0	0.0		
**Income status**	Income is less than expenses	*n*	57	7	0	2.364	0.80
		%	89.1	10.9	0.0		
	Income equals expenses	*n*	42	4	1		
		%	89.4	8.5	2.1		
	Income exceeds expenses	n	13	1	0		
		%	92.9	7.1	0.0		
**Person living together**	Alone	*n*	9	2	0	11.618	0.02*
		%	81.8	18.2	0.0		
	Spouse/family	*n*	102	8	1		
		%	91.9	7.2	0.9		
	Other	*n*	1	2	0		
		%	33.3	66.7	0.0		

## Discussion

Regarding the causes of death worldwide, it is observed that the leading cause is non-communicable diseases. Cancer, which falls within this scope, is considered a significant public health issue due to its increasing frequency in recent years [24]. The extension of life expectancy and advancements in early diagnosis and treatment options have led to an increase in cancer patients’ ED admissions. It is estimated that by the year 2030, approximately 70% of all cancers will occur in individuals aged 65 and older [25]. Studies investigating cancer patients’ visits to the emergency room show that visits were more common among adults aged 65 and over, and the average age was reported to vary between 61 and 76 [21, 26–30]. In our study, the average age was calculated as 70.92 ± 3.47 years.

The World Health Organization has stated that oncological diseases are more frequently observed in males from the age of 60 [26, 31]. In studies, the percentage of male participants are as follows: Koçak et al. [27], 58%; Bayrak and Kitis [21], 56.4%; Kerrouault et al. (2007) [32], 65%; and Olt et al. (2015) [33], 55.7%. In our study, the majority of participants were found to be male, consistent with the literature. These findings highlight the importance of tailoring nursing management and patient education programs to address the specific needs of male patients, particularly in understanding disease progression, treatment processes, and the psychosocial challenges they may face.

The three most common diagnoses of participants who applied to the ED were lung cancer (14.4%), breast cancer (13.6%), and stomach cancer (10.4%). The following results have been obtained for similar studies in the literature: In Koçak et al. [27], the three most common malignancies among were reported as cancer of the lung (30%), stomach (11%), and breast (11%). In Gallaway et al. [26] which examined cancer patients who applied to the ED, the three most common hemato-oncological diagnoses were cancer of the lung (13.0%), breast (9.9%), and colon or rectum (6.8%). In a study by Lee et al. [34], which investigated epidemiological trends in ED utilization for cancer-related issues, the most common cancer types observed during ED visits were reported as lung, liver, and colorectal. In a study by Rivera et al. [35], the most commonly reported types among cancer patients presenting to the ED were breast (14.9%), prostate (11.3%), and cancer (10.3%). The literature reveals that the majority of cancer patients presenting to the ED have gastrointestinal system cancer or respiratory system cancer [36–38]. In a study comparing general emergency services and those emergency services with comprehensive cancer centers, it was stated that those patients who most frequently applied to the latter type were those with hematological, breast, and gastrointestinal system cancers [39]. According to cancer statistics published in Turkey, the most common types of cancers are lung cancer for men, and breast cancer for women [11]. Thus, the results of our study appear largely compatible with the literature. Given these findings, the role of nursing becomes crucial in managing cancer patients’ educational needs. Tailored educational programs focusing on the specific needs and challenges associated with common cancer types can empower patients and their families while supporting continuity of care. It is important to provide individualized education to addressing patient concerns, and to coordinate care. In this way, nurses can help ensure that patients and their families are well-equipped to manage the complexities of cancer care, improving adherence to treatment plans and enhancing the overall patient experience.

Chemotherapy is one of the most common treatment methods for cancer-diagnosed patients. In our study, 82.4% of the participants stated that they received chemotherapy, which echoes findings in the literature [21, 40].

In our study, the most common reasons for emergency department (ED) visits among participants were infection, pain, nausea-vomiting, and dyspnea. These findings align partially with the literature. Koçak et al. [27] identified shortness of breath, abdominal pain, and deterioration in oral intake as the most frequent causes of ED visits, while Bayrak and Kitiş [21] reported pain, respiratory, and gastrointestinal issues. Vandyk et al. [41] highlighted neutropenic fever, infection, and nausea-vomiting as common reasons, whereas Barbara et al. [42] found pain to be the most prevalent cause. Similarly, Bozdemir et al. [36] emphasized pain, dyspnea, and nausea-vomiting as leading complaints. Other studies noted that cancer patients often visit EDs for neutropenic fever, electrolyte abnormalities, anemia, dehydration, and pneumonia [34, 35, 43, 44]. These findings underscore the multifactorial nature of ED admissions in oncology patients, particularly in managing treatment-related symptoms such as pain and nausea [45]. Given these challenges, nurse-led symptom management and patient education are critical in reducing emergency department (ED) visits. Nurses are uniquely positioned to implement proactive symptom management strategies, empowering patients with the knowledge required to recognize early signs of complications, manage treatment-related side effects, and engage in effective self-care practices. Educational interventions, particularly those targeting the management of symptoms such as pain and nausea, can enable patients to address these issues in the home setting, thereby reducing the frequency of unnecessary ED visits, and enhancing overall quality of life. This comprehensive, nursing-led approach plays a vital role in guiding patients through the complexities of cancer treatment and ultimately optimizing patient outcomes.

The high number of applications related to nausea/vomiting symptoms was associated with the fact that most participants received chemotherapy. The literature suggests that the reasons for admission are potentially less severe ones, and thus, education from oncology nurses for symptom control could reduce ED visits and increase disease self-management. The more frequent administration of chemotherapy and the need to control treatment-related side effects have increased the need for more accurate and more comprehensive patient and family education. The oncology nurse should comprehensively describe the potential side effects and issues caused by the use of antineoplastic agents, determine the likely timing of side effects, and assist patients in expressing their concerns [46]. The nurse should also provide symptom management to improve the individual and family’s quality of life. Additionally, the nurse should offer the individual and family information about the chemotherapy protocol, create an appropriate teaching plan which covers the needs of the patient and family regarding potential side effects of treatment, necessary self-care measures, and antiemetic regimen. This plan should be carefully implemented and evaluated [47].

In our study, only 39.2% of the participants stated that they were informed about situations requiring ED visits, and the majority mentioned being informed only about the possibility of fever. In the study by Bayrak and Kitiş [21], similar findings were reported. These results suggest that patients are generally informed about infection and the possibility of fever but may lack information about other potential side effects. The oncology nurse aims to improve the quality of life for individuals and families diagnosed with cancer and to ensure that they maintain their functionality at the highest level possible. Oncology nurses, who are always present with patients and have the opportunity to observe every aspect of their lives, play a crucial role with their knowledge and skills in controlling and managing patients’ symptoms. Particularly in the palliative care process, the oncology nurse should raise individuals and their families’ awareness about how to control symptoms at home and ensure the effective management of pain and other symptoms [48].

In the study, 89.6% were found to have visited the ED 1–3 times in the last year, and for 56%, the follow-up period was determined as 1–3 days. In another study examining the demographic and clinical characteristics of cancer patients who visited the ED, it was reported that the length of stay in the ED was generally less than 24 h [49]. However, there is only a limited number of studies focusing on cancer patients’ visits to the ED. Furthermore, it has been observed that the studies conducted did not specifically target the geriatric population but rather included all cancer patients. In this regard, our study makes a contribution to the existing literature. These results highlight the need for a specialized approach to managing oncology patients in the ED, particularly those over the age of 65. Nursing care for this population requires a comprehensive understanding of their unique challenges, such as comorbidities and potential side effects of cancer treatments. In geriatric oncology, nurses must play a key role in assessing patients’ clinical needs, coordinating with multidisciplinary teams, and providing appropriate follow-up care to prevent unnecessary return visits to the ED. Effective nursing management, including patient education and discharge planning, is critical to improving the quality of life and reducing hospital readmission rates for older cancer patients.

### Limits

The cross-sectional nature of the study, the small sample size, and the lack of variability in cancer types and drug protocols can be considered among the limitations of the study.

## Conclusion and recommendations

Advancements in the fields of medicine and technology have contributed to an increase in average life expectancy and the aging of the population, leading to an increased prevalence of non-communicable diseases, particularly cancer, along with challenges in managing treatment-related complications and symptoms. As a result, older oncology patients have experienced more frequent visits to the emergency department. In our study, we examined the reasons for and frequency of emergency department visits among oncology patients aged 65 and older, highlighting the importance of improving symptom management. Recurrent emergency department visits among cancer patients reveal insufficient knowledge about when to seek emergency care and gaps in post-treatment follow-up and palliative care. In this context, oncology nurses, particularly navigators, are expected to play a critical role in symptom management, early intervention, and care coordination. The establishment of a specialized care area for geriatric oncology patients would help address the unique needs of this population more effectively and improve the quality of healthcare services. Additionally, the development of evidence-based care guidelines for cancer patients is recommended, along with educational programs for healthcare professionals and caregivers to address these deficiencies.

## Data Availability

No datasets were generated or analysed during the current study.
